# Salicylic acid-induced differential resistance to the *Tomato yellow leaf curl virus* among resistant and susceptible tomato cultivars

**DOI:** 10.1186/s12870-019-1784-0

**Published:** 2019-05-02

**Authors:** Tong Li, Ying Huang, Zhi-Sheng Xu, Feng Wang, Ai-Sheng Xiong

**Affiliations:** State Key Laboratory of Crop Genetics and Germplasm Enhancement, Ministry of Agriculture and Rural Affairs Key Laboratory of Biology and Germplasm Enhancement of Horticultural Crops in East China, College of Horticulture, Nanjing Agricultural University, 1 Weigang, Nanjing, 210095 China

**Keywords:** Tomato yellow leaf curl virus, Salicylic acid, Ascorbic acid, ROS-scavenging enzymes, Systemic acquired resistance, Tomato

## Abstract

**Background:**

In higher plants, salicylic acid (SA) plays important roles in inducing resistance to biotic and abiotic stresses. *Tomato yellow leaf curl virus* (TYLCV) causes a highly devastating viral disease in plants, particularly in tomato. However, the roles of SA in inducing tomato plant resistance to TYLCV remain unclear.

**Results:**

In this study, we investigated whether the exogenous application of SA can improve the resistance of tomato plants to TYLCV in two tomato cultivars, resistant ‘Zhefen-702’ and susceptible ‘Jinpeng-1’. The impacts of SA on the accumulation of ascorbic acid (AsA) and biosynthetic gene expression, the activity of some important reactive oxygen species (ROS)-scavenging enzymes, and the expression patterns of stress-related genes were also determined. Results indicated that SA can effectively regulate the accumulation of AsA, especially in ‘Jinpeng-1’. Similarly, the expression patterns of most of the AsA biosynthetic genes showed a negative relationship with AsA accumulation in the resistant and susceptible tomato cultivars. In the two tomato cultivars, the activities of ascorbate peroxidase (APX) and peroxidase (POD) in the SA + TYLCV treated plants were increased during the experiment period except at 14 days (APX in ‘Jinpeng-1’ was also at 4 days) post infected (dpi) with TYLCV. Simultaneously, the activity of SOD was reduced in ‘Jinpeng-1’ and increased in ‘Zhefen-702’ after treatment with SA + TYLCV. SA can substantially induce the expression of ROS-scavenging genes at different extents. From 2 to 10 dpi, the virus content in the SA + TYLCV treated plants was remarkably lower than those in the TYLCV treated plants in ‘Jinpeng-1’and Zhefen-702’.

**Conclusions:**

The above results suggest that SA can enhance tomato plant resistance by modulating the expression of genes encoding for ROS-scavenging players, altering the activity of resistance-related enzymes, and inducing the expression of pathogenesis-related genes to produce systemic acquired resistance. Simultaneously, these results confirm that SA is a resistance-inducing factor against TYLCV infection that can be effectively applied in tomato plants.

**Electronic supplementary material:**

The online version of this article (10.1186/s12870-019-1784-0) contains supplementary material, which is available to authorized users.

## Background

Plant virus disease is a highly important crop disease and a great threat to plant growth and development. *Tomato yellow leaf curl virus* (TYLCV), which belongs to the genus *Begomovirus* [1], is a kind of plant virus disease that has a single-stranded DNA genome of 2.8 kb. This virus was first detected in China (Shanghai) in 2006 and presently occurs widely in China’s main tomato-producing areas [2, 3]. The main transmission route of the virus is *Bemisia tabaci* infection [4]. TYLCV can infect Solanaceae, Cucurbitaceae, and Piperaceae plants, such as tomato [5], cucumber [6], and pepper [7], and with the greatest harm to tomato. Because the virus is parasitic in plants, which lack an animal-like intact immune system, plants’ lives are endangered upon viral infection. The best way to manage TYLCV is to enhance host plant resistance against this virus.

In nature, plants are often simultaneously or sequentially attacked by numerous herbivorous insects and microbial pathogens (fungal, bacterial, and virus). In the long-term co-evolution process with pathogens, plants have gradually formed various mechanisms for resisting disease [8]. Induced resistance is an effective means to enhance plant disease resistance. A plant disease-inducing agent operates by stimulating the plant’s own defense mechanism to produce disease-resistance substances without exerting direct inhibitory effects on the pathogenic microorganisms. Plants can activate different types of induced resistance when infected by different pathogens [8]. Many non-biological factors, such as salicylic acid (SA), benzothiadiazole (BTH), and methyl jasmonate (MeJA), have been reported to induce plant resistance [9–11]. Induced resistance includes induced systemic resistance (ISR) and systemic acquired resistance (SAR) [12].

When plants are infected by pathogens, various physiological and biochemical changes occur to adapt or resist disease. Pathogenesis-related proteins (PRs) are a kind of potential disease-existent substances in plants. PRs can be produced or accumulated by plants when the latter are infected by pathogens or treated with certain compounds to manifest resistance against infections [13, 14]. Reactive oxygen species (ROS), such as hydrogen peroxide (H_2_O_2_), hydroxy1 radicals (HO), and superoxide anion radicals (O_2_^−^), are subsidiary products of cell metabolism [15]. The production and degradation of ROS in plants are in a state of balance under normal growth conditions [16]. The production amount of ROS is a stress reaction of plant to disease. The excessive accumulation of ROS can damage protein structure, induce protein fragmentation, and enhance membrane lipid peroxidation and result in irreversible damage and cell death [17, 18]. Two kinds of defense system exist in plants to induce resistance or repair the damage caused by the excessive accumulation of ROS derived from plant disease stress. One is a protective enzyme system that includes mainly ROS-eliminating enzymes, such as superoxide dismutase (SOD), guaiacol peroxidase (guaiacol POD), catalase (CAT), and ascorbate peroxidase (APX) [19, 20]. The other system is a non-enzyme system, of which ascorbic acid (AsA) and glutathione (GSH) are included. AsA, is one of the most abundant and efficient water-soluble antioxidant in plants, the molecule has critical roles in reducing ROS-induced oxidative damage caused by pathogens and environmental stress [21, 22]. The redox state of a plant changes when stressed by oxidation [23]. AsA eliminates excessive ROS in plants through the AsA–GSH cycle and redox state alteration [24].

SA is a phenolic compound produced by various plant species at different levels, this compound mediates many plant physiological processes, such as flowering [25, 26], seed germination [27], and induced plant resistance. The first reports describing salicylate function as disease resistance-inducing chemical are on the *Tobacco mosaic virus* in tobacco [28]. To date, many studies have identified that SA plays important roles as a signaling molecule in plant defensive responses to pathogens [29]. When a plant is infected by a pathogen, the SA content in the plant increases, and transduction of the SA signal activates the expression of genes encoding PR proteins [8]. For example, Matsuoka et al. identified that the untranslatable messenger RNA (mRNA) of a PR protein can be converted into a translatable state through the exogenous application of SA to tobacco [30]. Moreover, SA can regulate the ROS levels in plants by controlling the activity of protective enzymes and avoiding or eliminating the plant cell damage caused by oxygen stress. In tomato, the exogenous application of SA can increase phenylalanine ammonia lyase (PAL) and POD activities and induce and enhance tomato plant resistance to *Fusarium oxysporum* f. sp. *Lycopersici* (*Fol*) [31].

Tomato (*Solanum lycopersicum*), as a staple vegetable crop, is rich in nutrition and widely grown around the world. With the expanding area of tomato production and given the whitefly outbreak, the TYLCV has become a key factor restricting the production of tomato. The present work was carried out to investigate whether the exogenous application of SA can induce resistance in tomato plant when infected with TYLCV. The tomato plants of ‘Jinpeng-1’ and ‘Zhefen-702’, which are TYLCV susceptible and resistant tomato cultivars, were treated with SA by foliar spray and then inoculated with TYLCV. The contents of TYLCV virus, AsA, and dehydroascorbic acid (DHA) were measured. Furthermore, the effect of SA on the activities of antioxidant enzymes, the expression of stress-related gene, and those involved in AsA biosynthesis were also investigated.

## Results

### Phenotype and TYLCV virus content of tomato plants after treatment with SA and TYLCV

**Fig. 1:**
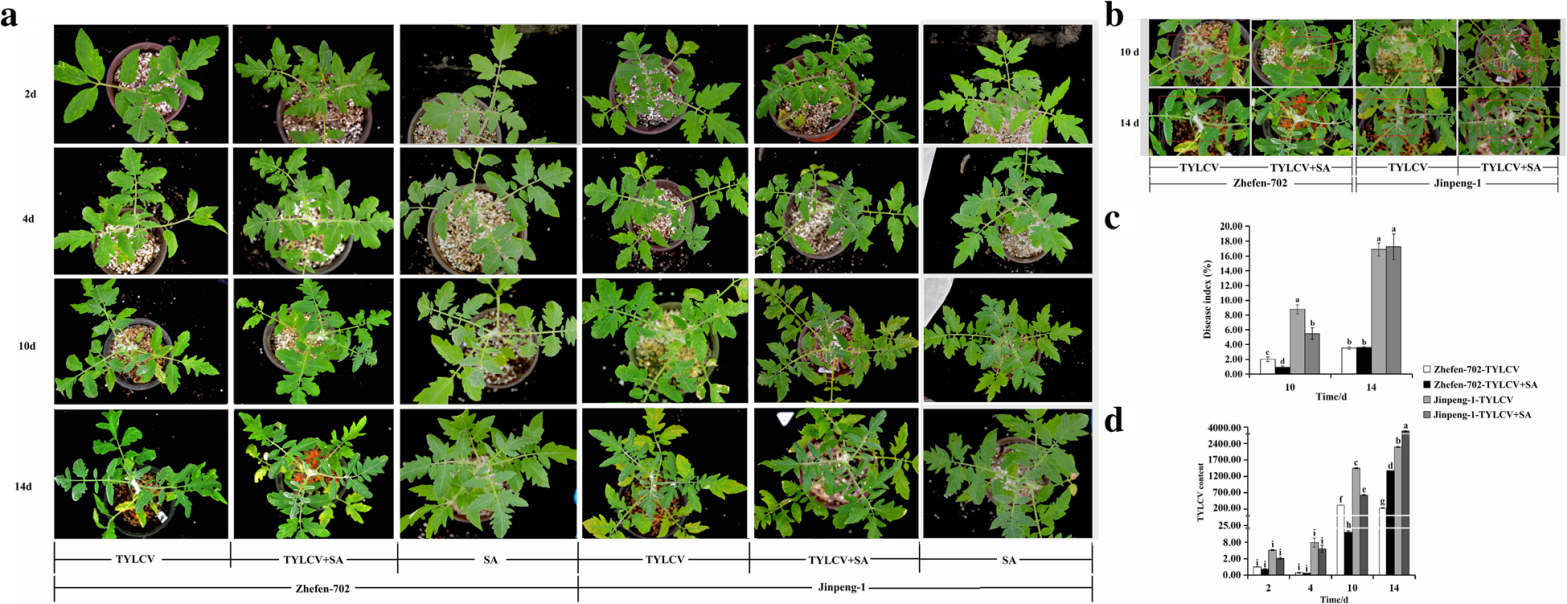
Effects of salicylic acid (SA) treatments and inoculation of TYLCV in tomato. **a** The phenotype of TYLCV, SA + TYLCV, and SA treated plants in two tomato cultivars after inoculated with TYLCV. The plants were first treated with SA through foliar spray, and then inoculated with TYLCV. SA treated plants was not infected by TYLCV. The photographs of the disease symptoms were taken at 2, 4, 10, 14 days post inoculated (dpi) with TYLCV. **b** Phenotype enlargement of SA + TYLCV and TYLCV treated plants in two tomato cultivars at 10 dpi and 14 dpi. **c** Disease index (DI) in SA + TYLCV and TYLCV treated plants; DI was measured at 10 dpi and 14 dpi. **d** The content of TYLCV virus in SA + TYLCV and TYLCV treated plants from two tomato cultivars. Values are means ±SD of three replicates

‘Jinpeng-1’ is susceptible, whereas ‘Zhefen-702’ is resistant, to TYLCV. The initial time of symptom appearance and disease development was later in ‘Zhefen-702’ than in ‘Jinpeng-1’ after TYLCV was inoculated [32, 33]. To determine whether SA induces tomato resistance against TYLCV, we treated tomato plants with 2 mM SA through foliar spray for 3 day (d) and then inoculated with TYLCV after 24 hours (h) of the last SA spraying. At 4 d after ‘Zhefen-702’ and ‘Jinpeng-1’ were infected with TYLCV, neither the only TYLCV nor the SA + TYLCV treated plants showed symptoms of the disease (Fig. 1a). Consistent with the symptom results, no significant differences in TYLCV virus content was noted between the TYLCV and SA + TYLCV treated plants, either between ‘Jinpeng-1’ and ‘Zhefen-702’ (Fig. 1d). Until 10 d post-inoculation (dpi) with TYLCV, the new leaves of the TYLCV treated ‘Jinpeng-1’ began to curl, but this symptom was not observed in the SA + TYLCV treated ‘Jinpeng-1’ and TYLCV treated ‘Zhefen-702’ (Figs. 1a, b). The disease index (DI) and TYLCV virus content of the TYLCV treated ‘Jinpeng-1’ was higher than those in ‘Zhefen-702’ (Figs. 1c, d). Whether in ‘Jinpeng-1’ or ‘Zhefen-702’, the DI and TYLCV virus content of the SA + TYLCV treated plants were considerably lower than those of TYLCV treated plants (Figs. 1c, d). At 14 dpi, all treated plants began to appear with symptoms induced by TYLCV (leaves yellowing, curling and shrinking), but the DI and TYLCV in ‘Zhefen-702’ were lower than that in ‘Jinpeng-1’. No substantial difference in DI was noted between the TYLCV treated and SA + TYLCV treated plants in either ‘Zhefen-702’ or ‘Jinpeng-1’, and the virus content in the SA + TYLCV treated plants was not lower than that in the TYLCV treated plants (Figs. 1c, d). In addition, SA treated plants exhibited a normal growth phenotype (Fig. 1a). These results indicated that the exogenous use of SA substantially decreased the accumulation of TYLCV virus in resistant and susceptible tomato cultivars, and this effect lasts for about 10 d.

### Changes in AsA content under TYLCV infection

The content of AsA and total AsA (T-AsA) in the three differently treated (SA, TYLCV, SA + TYLCV) tomato plants leaves were detected by HPLC (Additional file 1: Figure S1). From 2 to 10 dpi, the AsA content of only TYLCV treated plants was higher in ‘Zhefen-702’ than that in ‘Jinpeng-1’ (Table 1). In the two tomato cultivars, the T-AsA content of the plants treated with SA + TYLCV increased first, peaked at 4 dpi (the content was higher than that of TYLCV or SA treated) and then gradually decreased. In addition, the T-AsA content in the SA treated plants did not appear higher than that of TYLCV or TYLCV+SA treated plants both in ‘Zhefen-702’ and ‘Jinpeng-1’.

**Table 1:** Ascorbic acid (AsA), dehydroascorbic acid (DHA), and AsA/DHA ratio during the whole experiment period in two tomato cultivars

Cultivar		Zhefen-702			Jinpeng-1		
Time	Treatment	AsA (mg/g)	DHA (mg/g)	AsA/DHA	AsA (mg/g)	DHA (mg/g)	AsA/DHA
2 d	TYLCV	1.02 ± 0.01 b	0.92 ± 0.01 c	1.11 ± 0.03 e	0.00 ± 0.00 e	2.14 ± 0.08 a	0.00 ± 0.00 f
	SA + TYLCV	1.13 ± 0.03 ab	0.40 ± 0.02 e	2.78 ± 0.18 c	0.43 ± 0.04 d	1.34 ± 0.23 b	0.32 ± 0.02 ef
	SA	1.32 ± 0.45 a	0.16 ± 0.03 f	6.12 ± 0.98 a	0.88 ± 0.18 bc	0.23 ± 0.04 def	3.91 ± 0.06 c
4 d	TYLCV	1.27 ± 0.03 a	0.42 ± 0.04 e	3.05 ± 0.24 bc	1.03 ± 0.10 abc	0.88 ± 0.14 c	1.17 ± 0.08 def
	SA + TYLCV	1.20 ± 0.04 ab	0.64 ± 0.02 d	1.88 ± 0.13 d	0.83 ± 0.10 bc	1.41 ± 0.09 b	0.59 ± 0.03 ef
	SA	1.05 ± 0.17 b	0.33 ± 0.14 e	3.35 ± 0.92 b	0.47 ± 0.04 d	0.17 ± 0.02 ef	2.73 ± 0.48 cd
10 d	TYLCV	0.31 ± 0.08 c	1.23 ± 0.05 b	0.25 ± 0.08 f	0.00 ± 0.00 e	1.28 ± 0.13 b	0.00 ± 0.00 f
	SA + TYLCV	0.00 ± 0.00 c	1.48 ± 0.19 a	0.00 ± 0.00 f	0.49 ± 0.04 d	0.38 ± 0.01 de	1.28 ± 0.15 def
	SA	0.04 ± 0.02 c	1.25 ± 0.33 b	0.04 ± 0.01 f	1.11 ± 0.10 a	0.12 ± 0.01ef	9.00 ± 0.00 a
14 d	TYLCV	0.00 ± 0.00 c	1.33 ± 0.06 ab	0.00 ± 0.00 f	0.87 ± 0.08 bc	0.23 ± 0.01 def	3.82 ± 0.43 c
	SA + TYLCV	0.08 ± 0.04 c	0.78 ± 0.08 cd	0.10 ± 0.04 f	0.51 ± 0.13 d	0.08 ± 0.02 f	6.18 ± 0.15 b
	SA	0.15 ± 0.11 c	0.95 ± 0.01 c	0.16 ± 0.23 f	1.06 ± 0.05 ab	0.48 ± 0.02 d	2.18 ± 0.03 cde

The ratio of AsA to DHA showed different trend in the two tomato cultivars. In ‘Zhefen-702’, AsA/DHA ratio in SA treated plant was continued decline from 2 to 14 dpi. In TYLCV treated plant, it increased from 2 to 4 dpi then gradually decreased. In the plant treated with TYLCV+SA, the trend of AsA/DHA ratio was consistently increased first and then decreased. In ‘Jinpeng-1’, the ratio of AsA to DHA in TYLCV+SA treated plant was increased continuously. During the entire experimental period, except at 4 dpi, the ratio of AsA/DHA of TYLCV+SA treated plants was higher than that of TYLCV treated plants. In addition, the ratio of AsA to DHA in SA treated plants was higher than TYLCV treated plants in the entire treatment cycle except at 14 dpi.

### Changes in expression levels of genes involved in AsA biosynthesis in tomato plants

**Fig. 2:**
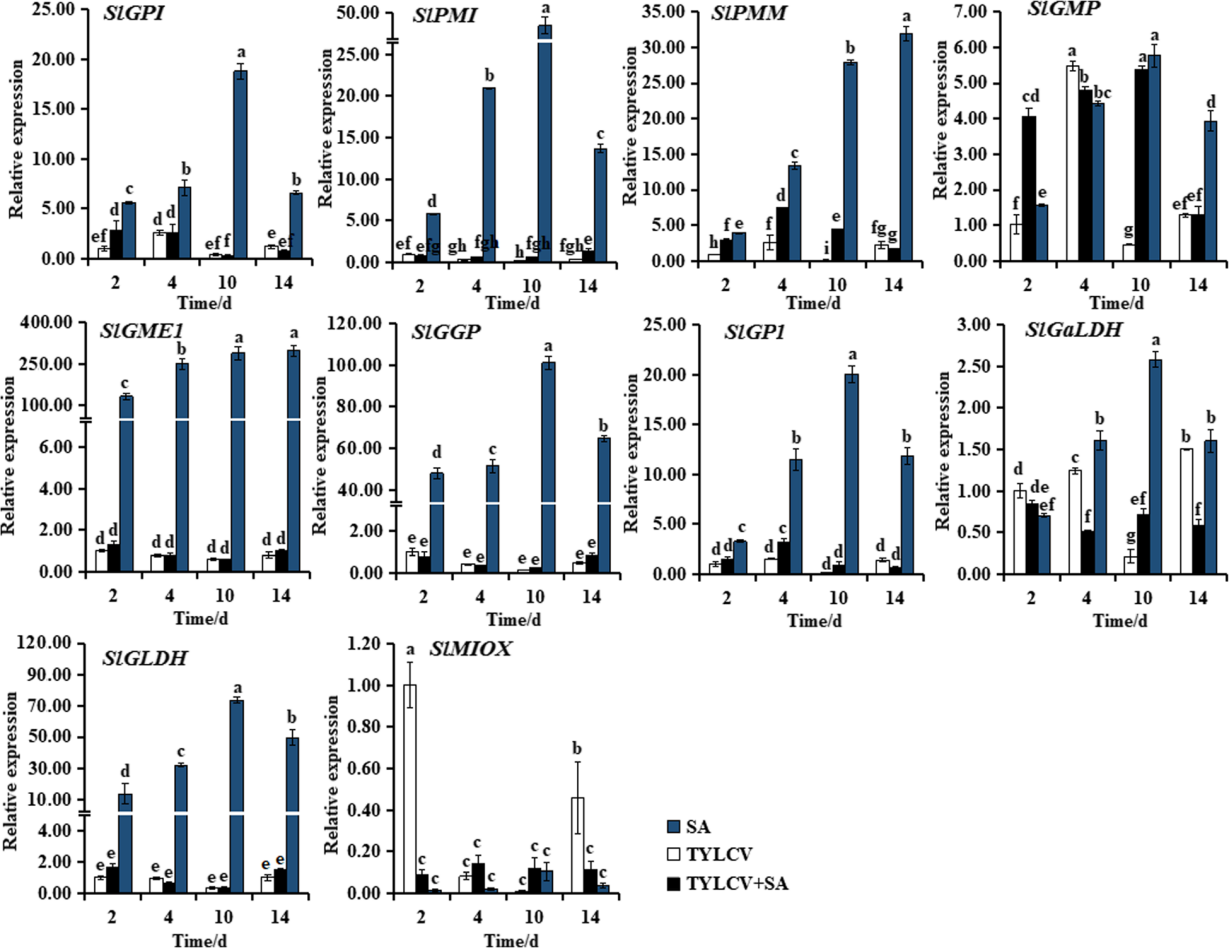
Expression patterns analyses of ascorbic acid (AsA) biosynthetic genes in salicylic acid (SA), TYLCV, and SA + TYLCV treated plants in ‘Zhefen-702’. The specific primer of *SlGPI*, *SlPMI*, *SlPMM*, *SlGMP*, *SlGME1*, *SlGGP*, *SlGP1*, *SlGaLDH*, *SlGLDH*, and *SlMIOX* were showed in Table 2. Values are means ±SD of three replicates

**Fig. 3:**
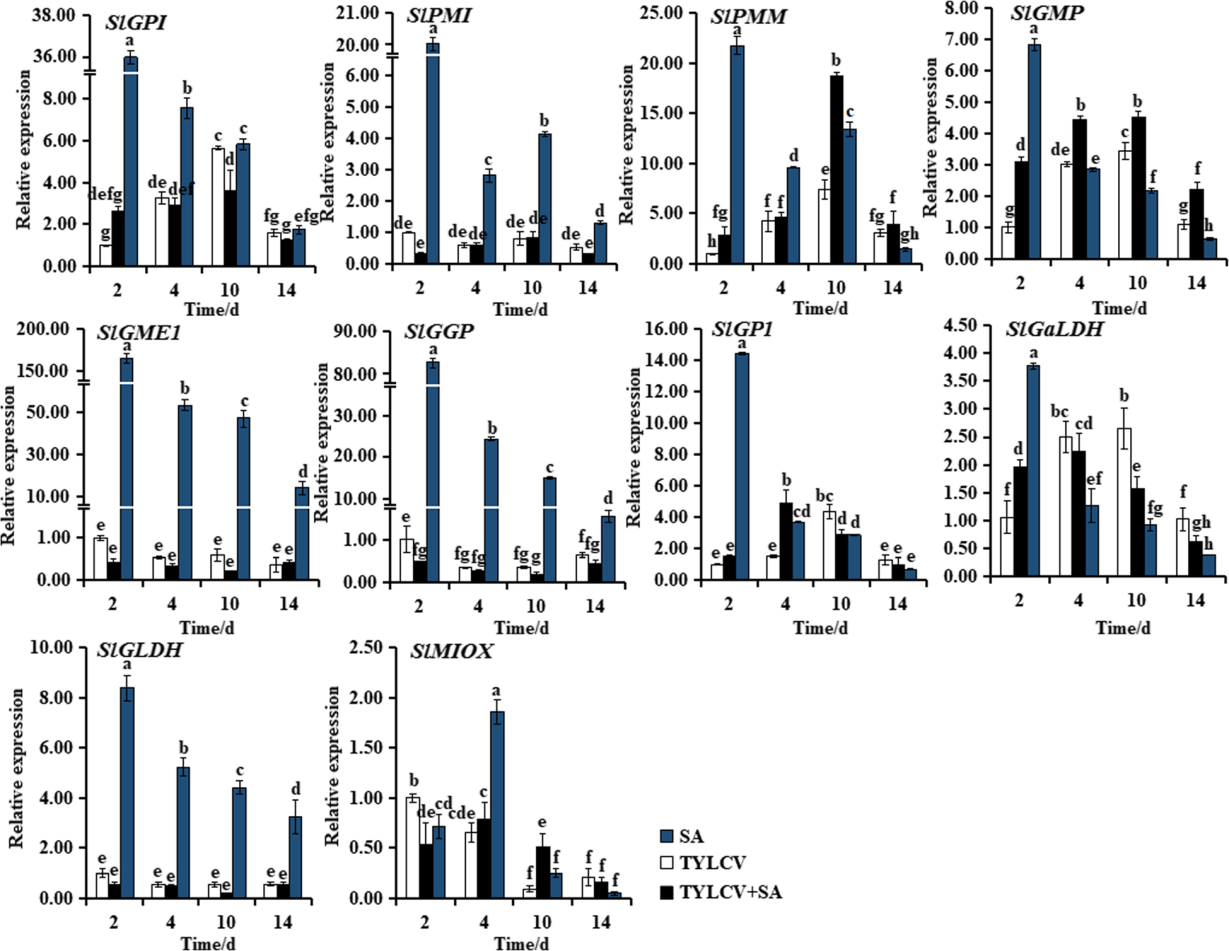
Expression patterns analyses of ascorbic acid (AsA) biosynthetic genes in salicylic acid (SA), TYLCV, and SA + TYLCV treated plants in ‘Jinpeng-1’. The specific primer of *SlGPI*, *SlPMI*, *SlPMM*, *SlGMP*, *SlGME1*, *SlGGP*, *SlGP1*, *SlGaLDH*, *SlGLDH*, and *SlMIOX* were showed in Table 2. Values are means ±SD of three replicates

The expression levels of 10 genes involved in AsA biosynthesis pathway in different treatment plants were examined in ‘Zhefen-702’ (Fig. 2) and ‘Jinpeng-1’ (Fig. 3). In the two selected tomato cultivars, the expression levels of AsA biosynthesis genes were obviously induced by SA treated. In ‘Zhefen-702’, the expression of *SlGMP* in three treatments (SA, TYLCV, SA + TYLCV) plants were increased from 2 to 10 dpi, then decreased. And the expression level of *SlGMP* in SA + TYLCV or SA treated plants was significantly higher than that in TYLCV treated plants during whole experiment time except at 4 dpi. From 4 to 10 dpi, the transcription levels of *SlPMI*, *SlPMM*, and *SlGP1* in SA + TYLCV treated plants were higher than that in TYLCV treated plants (Fig. 2). In ‘Jinpeng-1’, the expression level of *SlGaLDH* was obviously higher in TYLCV treated plants than that in SA + TYLCV treated or SA treated plants from 4 to 14 dpi. During the whole experiment time, the expression level of *SlGMP* in TYLCV+SA treated plants was the highest among three differently treated plants. The relative expression level of *SlMIOX* was significantly induced by SA from 4 to 10 dpi in TYLCV infected plants. And from 2 to 14 dpi, *SlPMM* hold a higher expression in SA + TYLCV treated plants. In SA treated plants, the expression of *SlGGP*, *SlGLDH*, *SlGME1*, *SlGP1*, *SlGaLDH*, and *SlGMP* were all increased at 2 d, and then decreased (Fig. 3).

### Changes in expression levels of genes involved in AsA recycling in tomato plants

**Fig. 4:**
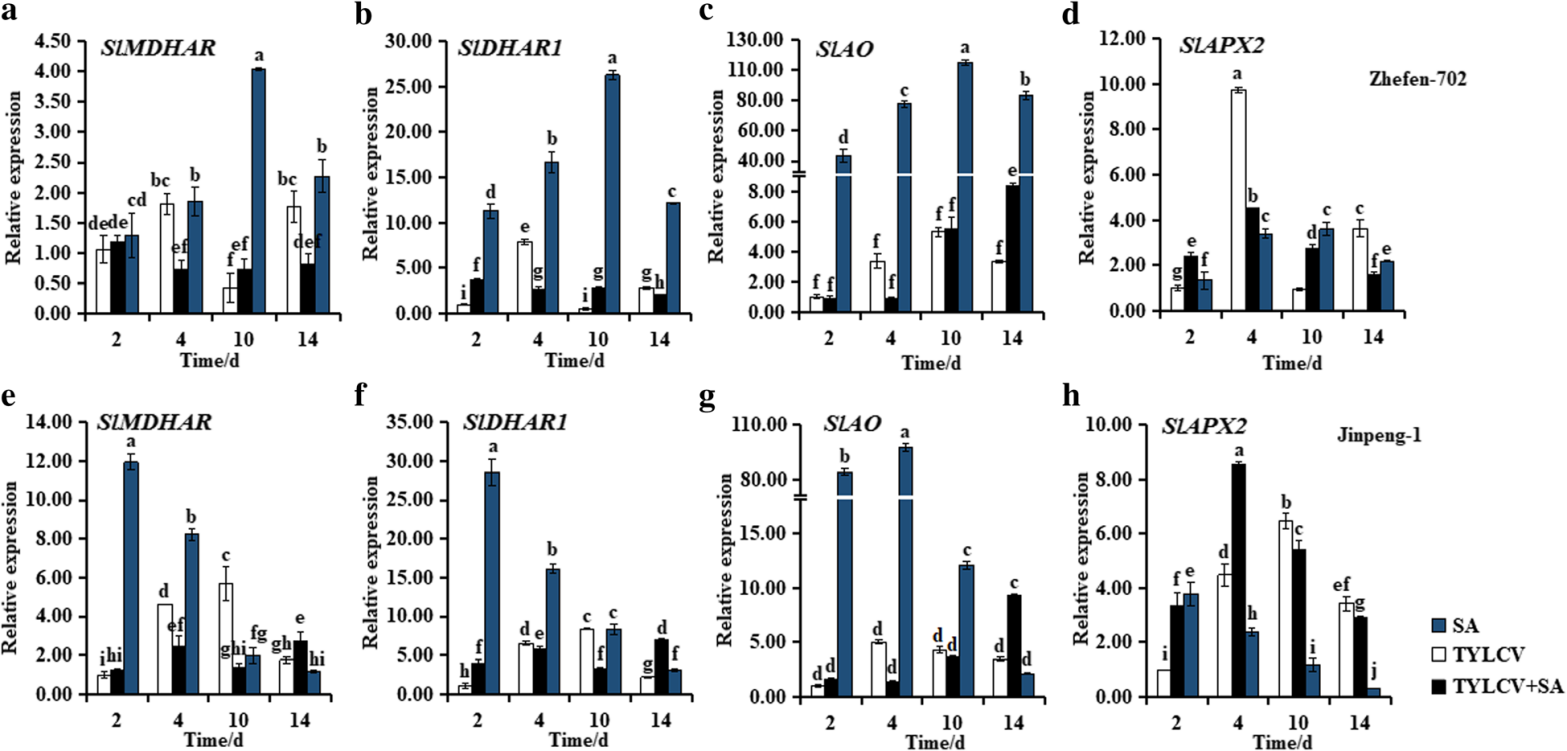
Expression patterns analyses of ascorbic acid (AsA) recycling genes in salicylic acid (SA), TYLCV, and SA + TYLCV treated plants in two tomato cultivars. **a**, (**b**), (**c**), and (**d**) are the expression patterns of *SlMDHAR*, *SlDHAR1*, *SlAO*, and *SlAPX2* in ‘Zhefen-702’, respectively. **e**, (**f)**, (**g**), and (**h**) are expression patterns of these genes in ‘Jinpeng-1’. The specific primer of *SlMDHAR*, *SlDHAR1*, *SlAO*, and *SlAPX2* were showed in Table 2. Values are means ±SD of three replicates

The transcription levels of 4 genes involved in AsA recycling were measured in tomato plants leaves by RT-qPCR (Fig. 4). In ‘Zhefen-702’, during the whole treatment cycle, the expression levels of *SlMDHAR*, *SlDHAR1*, and *SlAO* were induced by SA treated (Figs. 4a-c). Additionally, the transcription levels of *SlMDHAR* in SA + TYLCV treated plants exhibited a lower expression levels at 4 and 14 dpi in ‘Zhefen-702’, and at 4 and 10 dpi in ‘Jinpeng-1’ as compared with TYLCV infected plants (Figs. 4a, e). From 2 to 10 dpi, the trend of *SlAPX2* expression in TYLCV treated plants was similar with that in SA + TYLCV treated plants. And during this time, the transcription level of *SlAPX2* in SA + TYLCV treated plants was higher than that in TYLCV treated plants (Fig. 4d).

In ‘Jinpeng-1’, expression levels of *SlDHAR1* and *SlAO* were higher at 2 and 14 dpi, lower at 4 and 10 dpi in SA + TYLCV treated plants than TYLCV infected plants (Figs. 4f-g). *SlAPX2* in TYLCV and SA + TYLCV treated plants increased from 2 to 4 dpi, while the expression level was higher in SA + TYLCV treated plants. From 10 to 14 dpi, the expression level of *SlAPX2* in SA + TYLCV treated plants was lower than only TYLCV treated plants (Fig. 4h). In SA treated plants, expression of *SlMDHAR*, *SlDHAR1*, and *SlAPX* 2 were increased at 2 d, and then decreased (Figs. 4e-f, h).

### Changes in expression levels of stress-related genes in response to SA

**Fig. 5:**
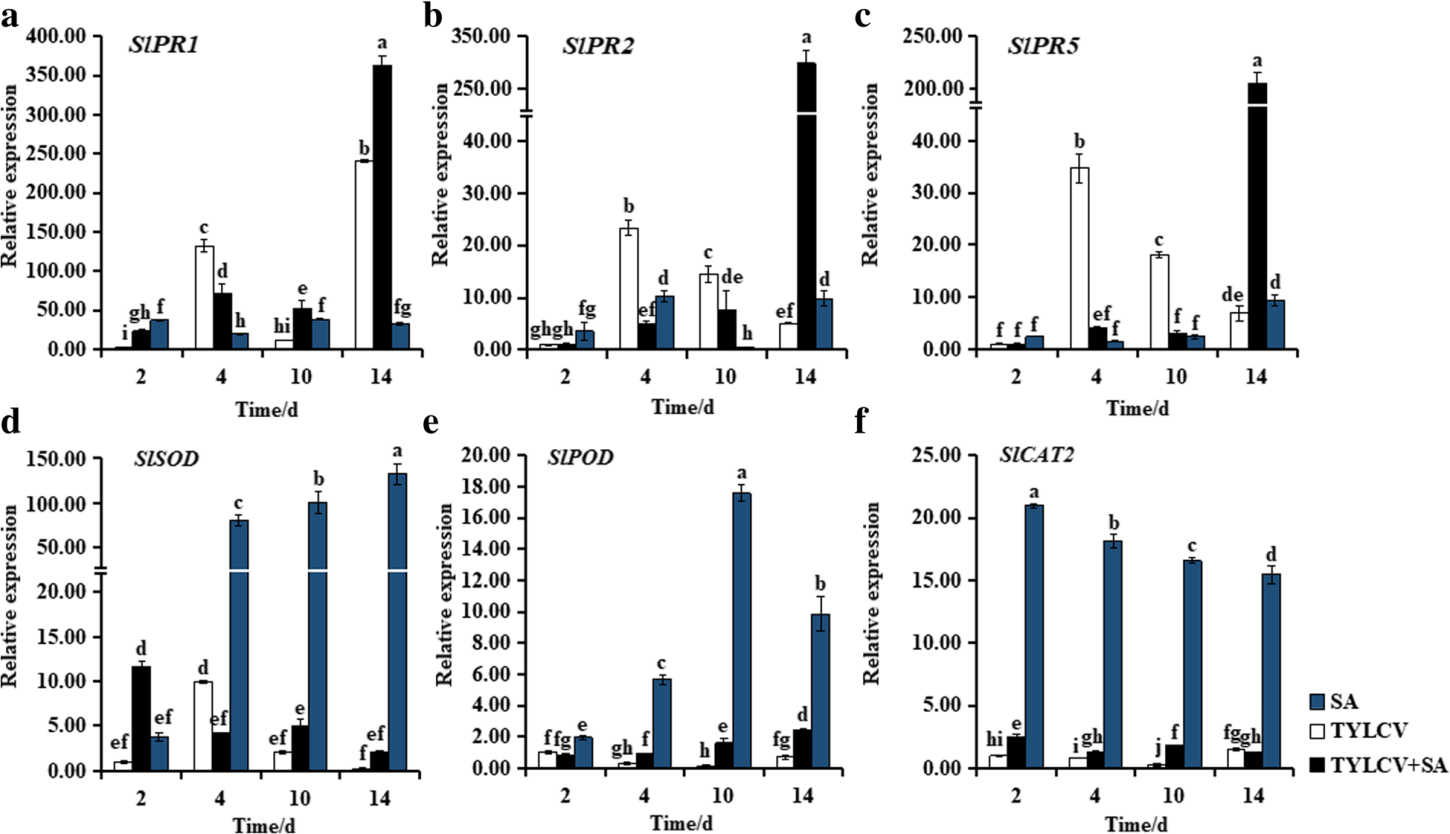
Expression patterns analyses of stress-related genes in salicylic acid (SA), TYLCV, and SA + TYLCV treated plants from ‘Zhefen-702’. The specific primers of (**a**) *SlPR1*, (**b**) *SlPR2*, (**c**) *SlPR5*, (**d**) *SlSOD*, (**e**) *SlPOD*, and (**f**) *SlCAT2* were showed in Table 2. Values are means ±SD of three replicates. Values are means ±SD of three replicates

**Fig. 6:**
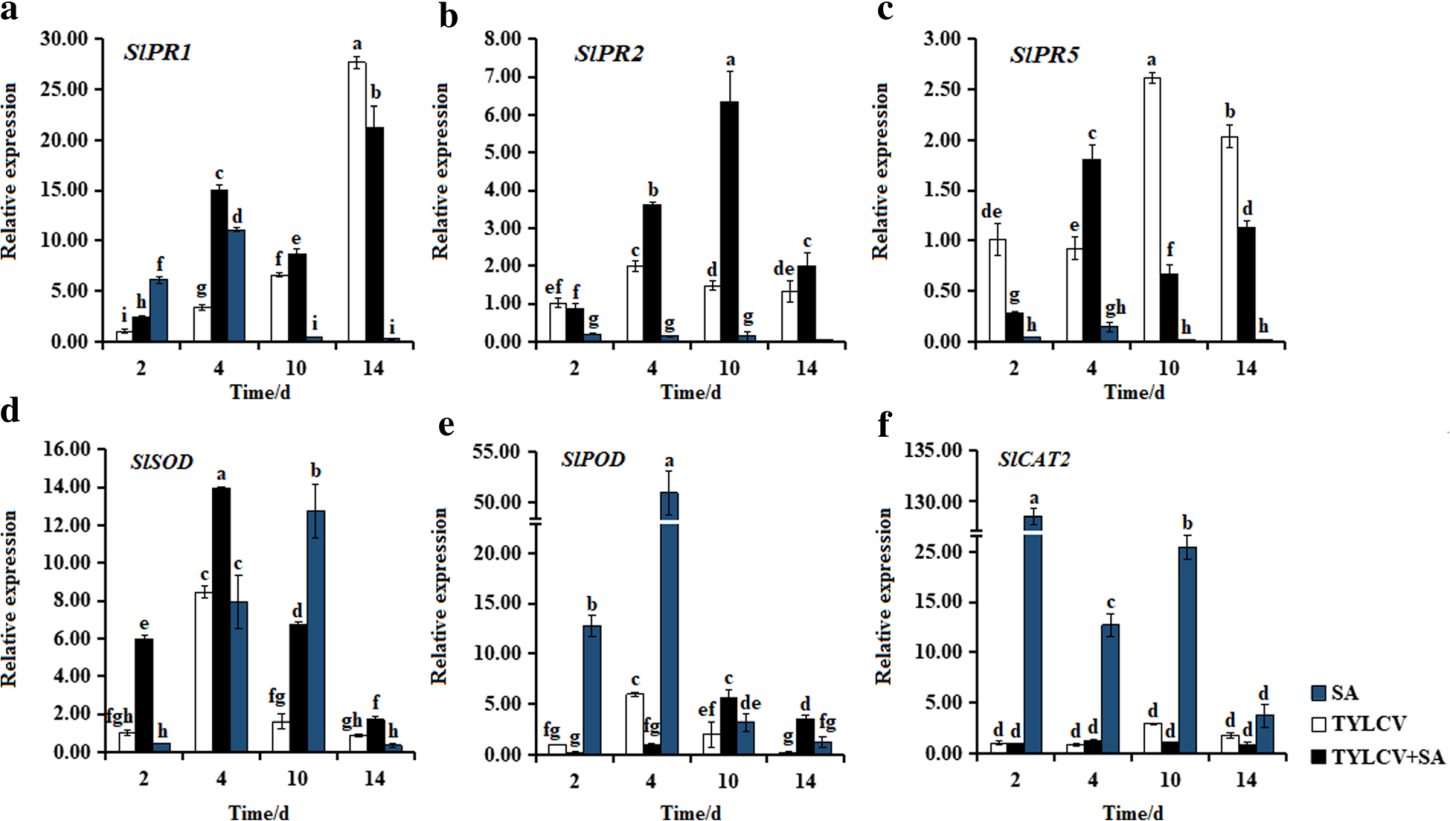
Expression patterns analyses of stress-related genes in salicylic acid (SA), TYLCV, and SA + TYLCV treated plants from ‘Jinpeng-1’. The specific primers of (**a**) *SlPR1*, (**b**) *SlPR2*, (**c**) *SlPR5*, (**d**) *SlSOD*, (**e**) *SlPOD*, and (**f**) *SlCAT2* were showed in Table 2. Values are means ±SD of three replicates. Values are means ±SD of three replicates

To further analyze the role of SA in induced resistance against TYLCV infection in tomato plants, we examined the expression levels of 6 stress-responsive genes that including *SlPR1*, *SlPR2*, *SlPR5* (SA response PR genes), *SlPOD*, *SlSOD*, and *SlCAT2* (encoding ROS scavenging enzyme) [34–36]. The expression levels of *SlPR1* in ‘Zhefen-702’ and ‘Jinpeng-1’ were induced by TYLCV, SA, and SA + TYLCV treatments. In ‘Zhefen-702’, *SlPR1* (Fig. 5a) and *SlSOD* (Fig. 5d) still remained at higher levels in SA + TYLCV treated plants than that in only TYLCV infected plants during whole treatment time except at 4 dpi. *SlPOD* (Fig. 5e) was remarkably induced by SA from 2 to 14 dpi. Similarly, the transcription level of *SlPR1* was higher in SA + TYLCV treated plants than only TYLCV infected plants in ‘Jinpeng-1’ from 2 to 10 dpi (Fig. 6a). Meanwhile, in ‘Jinpeng-1’, at 4 dpi, induced *SlPR1*, *SlPR2*, *SlPR5*, and *SlCAT2* mRNA accumulation in SA + TYLCV treated plants were increased and higher than that in TYLCV infected plants (Figs. 6a-c, f). The trend of *SlSOD* expression level in SA + TYLCV treated plants was similar with that in TYLCV infected plants. In addition, treated with SA, the expression level of *SlSOD* was induced from 2 to 10 dpi (Fig. 6d). The expression levels of *SlSOD*, *SlPOD*, and *SlCAT2* were induced in SA treated plants both in ‘Zhefen-702’ and ‘Jinpeng-1’ (Figs. 5d-f and 6d-f).

### Activity of antioxidant enzymes

**Fig. 7:**
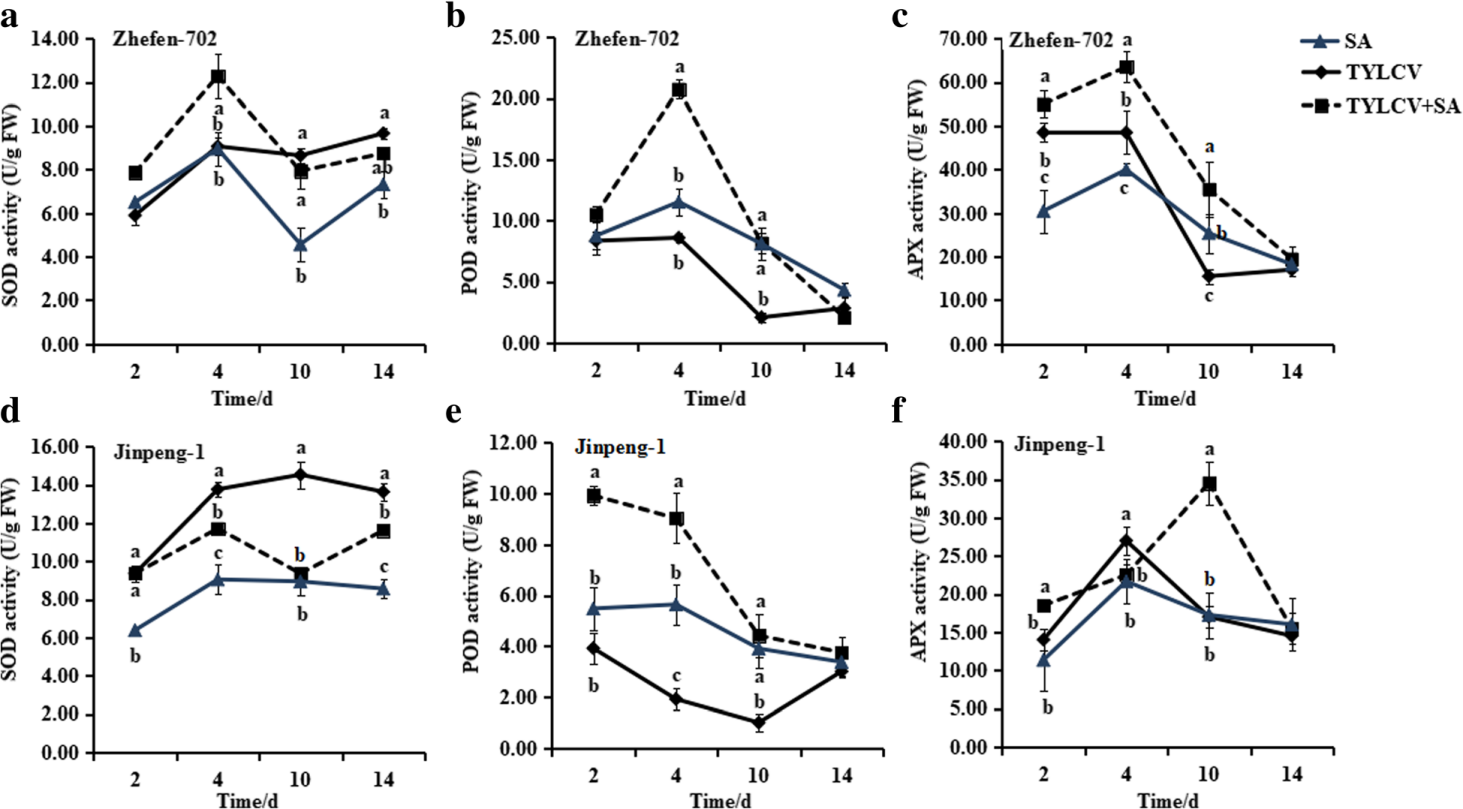
The activity of reactive oxygen species (ROS)-scavenging enzymes in salicylic acid (SA), TYLCV, and SA + TYLCV treated plants leaves. **a**, (**b**), and (**c**) are the activities of superoxide dismutase (SOD), peroxidase (POD), and ascorbate peroxidase (APX) in ‘Zhefen-702’, respectively. **d**, (**e**) and (**f**) are these ROS-scavenging enzymes in ‘Jinpeng-1’. The plants were first treated with SA through foliar spray, and then inoculated or non-inoculated with TYLCV. The leaves were harvest after inoculated or non-inoculated with TYLCV 2, 4, 10, 14 days and detected the activities of SOD, POD and APX. Values are means ±SD of three replicates

SA treatment, TYLCV infection, and SA + TYLCV treatment increased the activity of SOD. The activity of SOD in SA + TYLCV treated ‘Zhefen-702 ‘plants was significantly higher than other treatments from 2 to 4 dpi (Fig. 7a). In ‘Jinpeng-1′, SOD in TYLCV infected plants was highest during all experiment times among the treatments with SA, TYLCV, and SA + TYLCV (Fig. 7d).

In addition, as shown in Fig. 7b, we observed that the activity of POD enzyme was obviously increased after infected with TYLCV, and then decreased in ‘Zhefen-702’. The change trends of POD activity in plants treated with SA, TYLCV, and SA + TYLCV were similar. The SA + TYLCV treated plant has higher POD activity than other two treatment plants both in ‘Zhefen-702’ and ‘Jinpeng-1’ from 2 to 10 dpi, and there was no significant difference in POD activity between the three treatment plants at 14 dpi. The APX enzyme activity in ‘Zhefen-702’ was significantly higher than that in ‘Jinpeng-1’ (Figs. 7c, f). The activity of APX enzyme was increased after SA, TYLCV, or SA + TYLCV treatment. The most obvious effect of SA + TYLCV treatment on increasing enzyme activity was 2.36-fold and 2.02-fold of TYLCV infected plants in ‘Zhefen-702’ and ‘Jinpeng-1’ at 10 dpi, respectively. After infected with TYLCV, the activities of APX and POD were higher in ‘Zhefen-702’ when compared with these enzymes in ‘Jinpeng-1’, whereas the SOD activity showed opposite results.

## Discussion

Plant induced disease resistance is a ubiquitous genetic function in plants. More and more studies identified that enhanced plants resistance through induced by biological or chemical factors is one of the most effective way to control plant disease [31, 37, 38]. SA is a kind of chemical induce factor, and it’s an essential signaling molecule in SAR signals transduction pathways. As an induce factor, SA could active plants defense mechanism through affecting the physiological and biochemical status of plants such as increasing activity of disease-related enzymes and inducing the expression of PR-protein encoding genes [31, 39, 40]. In this study, we demonstrated that exogenous SA could induced tomato resistance to TYLCV infection within a certain times in TYLCV-resistant and susceptible tomato cultivars.

### SA treatment affects TYLCV virus accumulation and symptoms induced by TYLCV in tomato

SA treatment could induce resistance to virus disease in tomato [41]. When different TYLCV-resistant tomato varieties were inoculated with TYLCV, the synthesis of SA in the resistant varieties was significantly activated, and the SA responsive gene *PR1* was induced to express [42]. Therefore, we hypothesized that SA may affect the resistance of tomato plants to TYLCV. In order to verify this hypothesis, in this study, we applied exogenous application of SA to tomato varieties with different TYLCV-resistance, and inoculated with TYLCV. Our results showed that the accumulations of virus in the resistant or susceptible tomato varieties were reduced within a certain period of time. Additionally, in ‘Zhefen-702’ and ‘Jinpeng-1’, after SA treated, the expression of *SlPR1* was initially induced, and subsequent induction was not obvious. After treatment with TYLCV, the expression of *SlPR1* gradually increased with the increase of vaccination time. The induction effect was particularly obvious after treatment with SA + TYLCV. The difference in induction effect may be due to the fact that endogenous SA synthesis in plants is not activated when TYLCV is not inoculated. Exogenously applications of SA maybe function within a certain time. When infected with TYLCV, SA synthesis in plants was activated. As the inoculation time increased, the level of endogenous SA increased continuously, and more *SlPR1* gene expression was induced. Simultaneous treatment with SA and TYLCV, both endogenous and exogenous SA induce the expression of *SlPR1*, which increases the resistance of plant and reduces the accumulation of virus. Tomato plants infected with TYLCV virus will take a certain time to present the symptom phenotype [32, 43]. It was found that the leaf curl symptoms induced by TYLCV may be associated with the extreme down-regulation of the cellulose synthase family gene to decrease cellulose level [43]. Appropriate SA concentration could increase the cellulose content in rice [44]. In our study, TYLCV treated plants in ‘Jinpeng-1’ began to develop symptoms at 10 dpi, whereas the SA + TYLCV treated plants were not presented. Our results appear showed that exogenous SA reduce leaf curl induced by TYLCV infection may be responsible for increasing the content of cellulose. At 14 dpi, the TYLCV virus content in SA + TYLCV treated plants was not lower than that in TYLCV treated plants either in ‘Zhefen-702’ and ‘Jinpeng-1’, but there were no significant differences in DI. When the plant has high disease tolerance, the virus content is not necessarily positively correlated with the disease symptoms [33]. These results indicate that SA increase the disease tolerance of tomato plants to TYLCV, and the resistance induced by exogenous application of SA can last for about 10 d.

### The relationship between gene expression and AsA accumulation under SA treatment

ROS are constantly produced both in the normal development of plants and under stress. In the normal metabolic processes, the production and removal of ROS in plants is maintain a balanced state, but the balance is broken and ROS accumulation is increased when stressed [16]. AsA is one of the most abundant antioxidants in the cell, and most of it in the chloroplast exists in deoxidation form under normal physiological conditions [45]. The AsA in plants is oxidized to monodehydro ascorbic acid (MDHA) under the action of APX (at the same time, the H_2_O_2_ is catalytic and then deoxygenized to H_2_O). MDHA is not very stable, and can be converted to DHA without the action of any enzyme, while in the role of monodehydroascorbate reductase (MDAR), MDHA again transformed into AsA.

To investigate whether the positive effects of SA on AsA accumulation, we measured the expression levels of AsA biosynthetic pathways genes, the content of AsA and DHA after treatments with SA, TYLCV, and SA + TYLCV, respectively. Accumulation of AsA metabolites is not always associated with the expression of their biosynthetic genes, most of these genes expression tend to be positively correlated with total AsA and DHA, negatively correlated with AsA [46, 47]. A similar result was observed in present study, for example, the expression of most AsA biosynthetic genes in three treatment plants were showed a decline trend in ‘Jinpeng-1’ from 10 to 14 dpi, and the accumulation of AsA were continued to decline. Particularly, the expression of some AsA biosynthetic genes were lower in only TYLCV treated and SA + TYLCV treated plants than that in only SA treated plants. In addition, Gest et al. identified that the *MDHAR* negatively regulates the AsA levels in tomato [48]. Expression of *MDHAR* showed a positive correlation for AsA content from stage1 to stage3 in tea cultivar ‘Anjibaicha’, whereas negatively correlated with AsA content from stag 1 to stag 2 in ‘Yingshuang’ and ‘Huangjinya’ [49]. The expression of *SlMDHAR* in the present study was negatively correlated with AsA levels from 2 to 14 dpi in ‘Zhefen-702’; however, *SlMDHAR* was positively and correlated with AsA levels at 2 and 14 dpi in ‘Jinpeng-1’ (Fig. 4a, Table 1). The CsAPX protein play a critical role in AsA recycling in tea leaves, and the expression level of *CsAPX* could induced by high and low temperature [50]. In our study, the expression level of *SlAPX* was also induced by SA + TYLCV treatment (Figs. 4d, h). These results suggested that SA can induce the expression of *SlMDHAR* and *SlAPX* to promote AsA recycling.

The changes in the antioxidant redox state is an important process in response to oxidative burst [51]. The accumulation of DHA in apoplast would active the arrest of cell division what occur in harsh conditions will weaken the growth of plants cell and improve survival [52]. Our results showed that a large amount of DHA accumulated in SA + TYLCV treated plants after infected with TYLCV 10 d in ‘Zhefen-702’ (Table 1). In *Arabidopsis*, the accumulation of AsA could induce by JA, and promote the AsA–GSH cycle, eliminate ROS rapidly, thereby enhance plants disease resistance [53]. Similarly, in ‘Jinpeng-1’, SA could promote the accumulation of AsA or promote the AsA–GSH cycle (marked by AsA/DHA) from 2 to 14 dpi, and the content of AsA in SA treated plants was higher than TYLCV infected plants except at 4 dpi (Table 1). The above results indicated that the change of AsA levels was the result of the collective function of genes in AsA biosynthesis rather than a single gene, and the different genes played different regulatory roles. Simultaneously, SA could regulate the AsA content or change the redox state of AsA through inducing the increase or decrease of the expression of related genes, and thus regulating the resistance of tomato plants to TYLCV.

### Increased activity of ROS eliminating enzymes under SA treatment

SOD is the first antioxidant enzyme in the process of ROS scavenging reaction, its main role is that it can rapidly disproportionated O_2_^−^ to H_2_O_2_ and molecular oxygen [54]. Widely found in plants, animals, and microorganisms, POD is a key antioxidant enzyme that clears H_2_O_2_ through catalyzing various redox reaction which H_2_O_2_ involved, H_2_O_2_ is deoxygenated to H_2_O thus reducing the internal oxidation state of plant [54]. APX is a kind of POD that uses AsA as an electron donor to remove H_2_O_2_. The antioxidant enzymes activities in susceptible cultivar were lower than tolerant cultivar after infected with *M. graminicola* in wheat [38]. In TYLCV susceptible cultivar ‘Jinpeng-1’, the activities of SOD was higher, POD and APX were weaker in only TYLCV treated plants than those in TYLCV resistant cultivar ‘Zhefen-702’ after infected with TYLCV (Fig. 7). Perhaps this result is one of the reasons for the different resistance between different resistant tomato cultivars.

Huang et al. [55] observed that when faced with TYLCV infection, APX protein were detected in both resistant and susceptible cultivars by comparative proteomics, and the transcription level of *APX* was decreased in the early stage of TYLCV infection but increased at 15 dpi in ‘Zheza-301’ (TYLCV-resistant cultivar), whereas it was increased first and decreased from 10 to 15 dpi in ‘Jinpeng-1’. It was found in this study that the trend of APX enzyme activity in ‘Zhefen-702’ and ‘Jinpeng-1’ was similar to the transcription level of *APX* that observed by Huang et al., and SA treated increased the activity of APX enzyme. Many studies indicate that SA could enhance the plants resistance by regulating the activities of peroxidase. In *Ya Li* pear trees, spray 2.5 mM SA were obvious increased the activities of POD and PAL, reduced the activities of APX and CAT, showed that SA could coordinately regulate the enzymes that exerting their functions in different ways and promote the protection of *Ya Li* pear fruit against postharvest disease [56]. Exogenous application of SA through root feeding and foliar spray increased the activities of PAL and POD, probably leading to enhance resistance of tomato plants to *Fol* [31]*.* In chickpea, sprayed with SA could induce an remarkable increase of POD and PPO enzyme activities, thus contributing in plants induction defensive system [37]. The same in the present study, the activities of POD and APX in two experiment tomato cultivars increased to a great extent in SA + TYLCV treated plants as compared with TYLCV and SA treated plants in the whole (Figs. 7b-c, e-f). On the contrary, the activity of SOD in SA + TYLCV treated plants was lower in ‘Jinpeng-1’ and higher in ‘Zhefen-702’, respectively (Figs. 7a, d). These results showed that SA probably induced tomato resistance of TYLCV through enhancing or inhibiting the activity of SOD, increasing the activities of POD and APX, as a result, the ROS scavenging system was maintained in balance.

### Inducing the expression of stress-related genes under SA treatment

PRs are a protein or proteins that produced or accumulated after plant infected with pathogen or treatment by some specific compound. PRs is a potential resistant substance in plants, which can resist the invasion of pathogens [57]. Induction of PRs had been taken as a marker of the induced state [13]. The endogenous SA content of plants would increase sharply after infected with pathogens, which can induced the expression of PR genes [8]. It was found in this study that the expression levels of *SlPR1*, *SlPR2*, and *SlPR5* were induced by TYLCV treatment in two tomato cultivars. Exogenous application of SA could induce the expression of PRs [39, 58, 59]. Our experiment results showed that the expression of *SlPR1*, *SlPR2*, and *SlPR5* were induced by SA + TYLCV treatment in two tomato cultivars (Figs. 5, 6). It has been described that SA induced tomato resistance to different RNA viruses appeared to be independent of PR protein, and Campos et al. found that SA could pre-induce RNA silencing-related genes to delay the accumulation of RNA pathogen [41, 60]. These differences indicate that in response to different types of viral diseases, SA induces resistance in host plants may through different modes of action. Additionally, we investigated the expression patterns of ROS-scavenging enzyme encoding genes in SA + TYLCV treated and TYLCV treated plants, the expression of *SlSOD*, *SlPOD*, and *SlCAT2* were all induced in SA + TYLCV treated plants. Previous researchers have found that regulating the defense response of tomato may be through regulating the expression of ROS-scavenging enzyme encoding genes and PRs-encoding genes, thus modulating ROS and SA-signaling pathway [36]. The above results indicated that SA could regulate the ROS and SA-signaling pathway through regulating the expression patterns of ROS related genes and PRs genes, respectively, thus contributing in enhanced resistance of tomato to TYLCV.

## Conclusion

In present study, two tomato cultivars ‘Zhefen-702’ (TYLCV-resistant) and ‘Jinpeng-1’ (TYLCV-susceptible) were sprayed three times continuous with 2 mM SA in the four-leaf stage, then infected with TYLCV. The effect of SA on the content of AsA and DHA, the activities of ROS-scavenging enzymes, and the expression patterns of genes which encoding ROS-scavenging enzymes and PRs were detected. Our results demonstrated that sprayed SA may enhance tomato plants resistance to TYLCV in resistant and susceptible tomato cultivars during a certain time base on the following two aspects. One is that SA could enhance the ability of plants to remove ROS through affecting the synthesis of AsA and increasing the activity of ROS eliminating enzymes; the other is SA probably induce tomato plants to produce SAR through inducing the expression of PRs. The results of this research indicated that SA may be used as a factor to induce resistance in tomato plants, and it provided a theoretical basis for the possibility of using inducer as a control method for the prevention and treatment of TYLCV. Further work is required to identify the interaction of SA with the ROS pathway by increasing the level of endogenous SA in tomato plants, and to characterize the SA-induced resistance network against TYLCV.

## Materials and methods

### Plant materials and growth condition

‘Jinpeng-1’ (susceptible to TYLCV infection) and ‘Zhefen-702’ (resistant to TYLCV infection) were used as experiment materials. The seeds of ‘Jinpeng-1’ and ‘Zhefen-702’ were obtained from Xi’an Jinpeng Seed Co., Ltd., and Zhejiang Academy of Agricultural Sciences, respectively. The seeds of different tomato cultivars were put in the plug containing soil, perlite, and vermiculite (2:1:1, v/v/v) mixture. The seedlings were moved into plastic pots when grew to four-leaf period. The whole growth stages of the plant were grown in a growth chamber under 12 h light (25 °C)/12 h dark (18 °C) cycle. The relative humidity was maintained at 60 to 70%.

### SA treatment and TYLCV infection

For SA treatment, the plant with the same growth of four-leaf stage were selected and divided into three groups. SA treated group, exogenous application of SA (2 mM) through foliar spray, and not infected with TYLCV; TYLCV treated group, plants were sprayed with deionized water, after 24 h of last deionized water spraying, the seedlings were transferred into a greenhouse to inoculate with TYLCV; SA + TYLCV treated group, exogenous application of SA (2 mM) through foliar spray, after 24 h of last SA spraying, the seedlings were transferred into a greenhouse to inoculate with TYLCV. Leaf spraying was carried out at the same time each day and repeated for 3 d. The process of TYLCV infection was described by Huang et al. [61]. After inoculated with TYLCV 2, 4, 10, 14 d, the leaves of plants were harvested for physiological parameter determination and gene expression detection. For each treatment, 40 seedlings of each tomato cultivars were used, and leaves from the three random plants of each group were collected at designated time points, immediately frozen in liquid nitrogen and stored at − 80 °C.

### Determination of TYLCV virus content and DI

The DNA of plant leaves which inoculated with TYLCV 2, 4, 10, 14 d, respectively was extracted. And we designed specific primer to detect the content of TYLCV virus. Specific primers are shown in Table 2. Symptoms were evaluated according to the visual symptom-severity scale described by Friedmann et al. [62] after some modifications: 0 = No visible symptoms apparent; 1 = the leaves are lightly yellowed or curled; 2 = about 10–30% of leaves are yellowed, the edge of the new leaf is yellowed; 3 = about 30–60% of leaves are yellowed, the edge of the new leaf is yellowed and curled; 4 = all leaves are yellowed and curled, new leaf is yellowed and curled, plant dwarfing. DI was calculated as the following formula: DI (%) = ∑ (disease severity **×** number of plants in that disease severity) **×** 100/ (total number of plants **×** highest scale).

**Table 2:** Primers used for RT-qPCR

Gene	Full name	Forward primer (5′-3′)	Reverse primer (5′-3′)
*SlGPI*	Glucose-6-phosphate isomerase	AGAGGGTTCGCAGTGGTTCCTGGGTT	CCTACCCAGTCCCAGAAAGCGAATGC
*SlPMI*	Phosphomannose isomerase	TGGACATGCTAATGGCGGAGGA	AACGAGTGGCTGCTCCTTATGC
*SlPMM*	Phosphomannomutase	TGTTGGAGTTGTTGGAGGTTCTGAC	CTCCCAATAGGCGACACATTTAGCA
*SlGMP*	GDP-D-mannose pyrophosphorylase	AAACCTGAAATCGTGATGTGAGA	TGAAGAAGAGGAGAACTGGAAAC
*SlGME1*	GDP-mannose 3′,5′-epimerase 1	TCCGACTTCCGTGAGCCAGTAA	CGTGTTGTCTGAGTTGCGACCA
*SlGGP*	GDP-L-galactose-1-phosphate phosphorylase 1	AGGCTTCTCGCAGAGGTCTCAC	CCATCAGTGCCGTCCTCGTTCT
*SlGP1*	L-Galactose-1-phosphate phosphatase 1	GTGGCATGTGGAAGGCTTGAACT	CGGCTACACGTTGTGATGTGATGT
*SlGaLDH*	L-Galactose dehydrogenase	ATGAGACACTTCCCGCCCTTCTAA	CTTCGTCCATTCCAGTCGTTGCTA
*SlGLDH*	L-Galactono-1,4-lactone dehydrogenase	TGCTCCGTTCCTTCGCTTCCA	TGGCGGCAGCAGAACCTAAGA
SlMIOX	Myo-Inositol oxygenase	GGGCTTCCTCAATGGGCTGTT	TGTCCTCCTCATTCATCAAGTGTGT
*SlMDHAR*	Monodehydroascorbate reductase	GCTGGTGGTGAGAGGCAAACA	GGCAAGAGAAGGAGTGAACAGTCT
*SlDHAR1*	Dehydroascorbate reductase 1	AGCAGGCTCTCCTTGATGAACTAA	TCAGGCACACTCCACTTCTTGAA
*SlAO*	L-ascorbate oxidase	CAGGATGGCTCAGAGTGTTGCTAT	GGCGATCAGGTAAGGCGTATGG
*SlAPX*	Ascorbate peroxidase	TGGGAGGGTGGTGACATATTTT	TTGAAGTGCATAACTTCCCATCTTT
*SlPR1*	Pathogenesis-related protein	GTGTCCGAGAGGCCAGACTA	ATTGTTGCAACGAGCCCGA
*SlPR2*	Beta-1,3-glucanase	TCCAGGTAGAGACAGTGGTAAA	CCTAAATATGTCGCGGTTGAGA
*SlPR5*	Thaumatin-like protein	GGCCCATGTGGTCCTACAAA	GGCAACATAGTTTAGCAGACCG
*SlSOD*	Superoxide dismutase	CTTCACCACAACCAGCACTACCAA	TCCAGGAGCAAGTCCAGTTATACGA
*SlPOD*	Peroxidase	TTGGAGTGTCTCGTTGCTCA	TTCACCAGCACTCCCTGTCT
*SlCAT2*	Catalase	CAAGTTCGCCATGCTGAGGTGTAT	AGCCTGAGACCAGTATGTGATCCAA
*Tubulin*	*Tubulin*	TGACGAAGTCAGGACAGGAA	CTGCATCTTCTTTGCCACTG
*TYLCV*	TYLCV DNA detected	ATGTCGAAGCGACCAGGCGATATAAT	TTAATTTGATATTGAATCATAGAAAT

### Enzyme activity assays

The fresh leaves (0.2 g) of seedlings after TYLCV infection were ground in 1 mL of 50 mM ice-cold phosphate buffer (pH 7.8) and centrifuged at 12,000 rpm for 20 min at 4 °C. The supernatant were used for the determination of antioxidant enzyme activities. SOD and POD activity was measured following the method that described by Macadam [63]. The SOD activity was detected through determining the ability of this enzyme to inhibit the photochemical reduction of nitroblue tetrazolium (NBT). One unit of POD enzyme activity represented the change in absorbance by 1unit per minute under conditions of assay. The activity of APX was evaluated using the modified method described by Nakano and Asada [64]. One unit of APX activity was defined as the change in absorbance by 0.1 units per minute under conditions of assay. Three biological repeats were performed for each experiment.

### Analysis of AsA and Total-AsA

The content of AsA, DHA and T-AsA were assayed according to the method described by Melino [65] and Ren [46], with slight modifications. Briefly, the samples (0.2 g) of leaves were homogenized in 2 mL of ice-cold 0.1% (w/v) oxalic acid. The mixture was transferred to a 5 mL centrifuge tube and centrifuged at 12,000 rpm for 20 min at 4 °C. The supernatant was filtered by a 0.45 μm membrane syringe filter. Then the T-AsA was analyzed by adding 20 mg/L DTT (DL-Dithiothreitol) to the 500 μL extracts at a 1:1 ratio, and reaction for 15 min in the dark at room temperature. Finally, the sample was used for HPLC assays of AsA and T-AsA at a wavelength of 245 nm. The difference between T-AsA and AsA was the content of DHA. Three biological repeats were performed for each experiment.

### Gene expression analysis

Trizol reagent (TaKaRa, Dalian, China) was used to extract Total RNAs according to the manufacture’s protocols. Prime Script RT reagent kit (TaKaRa, Dalian, China) was used to convert total RNAs into cDNAs. The SYBR Premix *Ex Taq* kit (TaKaRa, Dalian, China) was used with for quantitative real-time PCR (RT-qPCR) analysis, 20 μL reaction mixture consisting of 10 μL SYBR Premix *Ex Taq*, deionized water (7.2 μL), diluted cDNA (2 μL), and 0.4 μL of each primer (Table 2). The program of the RT-qPCR was as follows: 95 °C for 30 s initially, followed by 40 cycles at 95 °C for 5 s; 60 °C for 30 s and melting curve analysis (61 cycles) at 65 °C for 10 s. The *Tubulin* acted as the internal reference [66], and the RNA level were calculated based on the 2^-ΔΔCT^ method [67]. Three biological repeats were performed for each experiment.

### Statistical analysis

Three independent biological repetitions of each group (SA, TYLCV, and SA + TYLCV) were used for all described experiments. The results were expressed as mean ± standard deviation (SD). Each experiment was repeated at least three times with similar results. Statistical analysis was performed by one-way analysis of variance (ANOVA) in SPSS 20.0 software and the statistical difference were detected based on Duncan’s multiple range test at a 0.05 probability.

## Additional file

Additional file 1: **Figure S1.** HPLC chromatogram of ascorbic acid (AsA) in two tomato cultivars at 4 days after infected with TYLCV (a), (e) The standard curve of AsA. (b), (c), (d) The HPLC chromatogram of AsA in salicylic acid (SA), TYLCV, and SA + TYLCV treated plants in ‘Zhefen-702’ at 4 days post inoculated (dpi) with TYLCV. (f), (g), (h) The HPLC chromatogram of AsA in SA, TYLCV, and SA + TYLCV treated plants in ‘Jinpeng-1’ at 4 dpi. **Figure S2.** Phenotype enlargement of SA + TYLCV and only TYLCV treated plants in ‘Zhefen-702’ during whole experiment period. **Figure S3.** Phenotype enlargement of SA + TYLCV and only TYLCV treated plants in ‘Jinpeng-1’ during whole experiment period. (DOCX 2033 kb)

## Abbreviations

APX: Ascorbate peroxidase
AsA: Ascorbic acid
BTH: Benzothiadiazole
CAT: Catalase
DHA: Dehydroascorbic acid
GSH: Glutathione
H_2_O_2_: Hydrogen peroxide
HO: Hydroxy1 radicals
HR: Hypersensitive response
ISR: Induced systemic resistance
MeJA: Methyl jasmonate
O_2_^−^: Superoxide anion radicals
PAL: Phenylalanine ammonia lyase
POD: Peroxidase
PRs: Pathosgenetics-related proteins
ROS: Reactive oxygen species
RT-qPCR: Quantitative real-time polymerase chain reaction
SA: Salicylic acid
SAR: Systemic acquired resistance
SOD: Superoxide dismutase
T-AsA: Total ascorbic acid
TYLCV: Tomato yellow leaf curl virus
