# Supplementary material for: Salicylic acid-induced differential resistance to the Tomato yellow leaf curl virus among resistant and susceptible tomato cultivars
Source: BMC Plant Biol. 2019 May 2;19:173. doi: 10.1186/s12870-019-1784-0 (PMC6498608; doi:10.1186/s12870-019-1784-0)
Supplement: Supplementary file 1 — Figure S1. HPLC chromatogram of ascorbic acid (AsA) in two tomato cultivars at 4 days after infected with TYLCV (a), (e) The standard curve of AsA. (b), (c), (d) The HPLC chromatogram of AsA in salicylic acid (SA), TYLCV, and SA + TYLCV treated plants in ‘Zhefen-702’ at 4 days post inoculated (dpi) with TYLCV. (f), (g), (h) The HPLC chromatogram of AsA in SA, TYLCV, and SA + TYLCV treated plants in ‘Jinpeng-1’ at 4 dpi. Figure S2. Phenotype enlargement of SA + TYLCV and only TYLCV treated plants in ‘Zhefen-702’ during whole experiment period. Figure S3. Phenotype enlargement of SA + TYLCV and only TYLCV treated plants in ‘Jinpeng-1’ during whole experiment period. (DOCX 2033 kb) [file 12870_2019_1784_MOESM1_ESM.docx]

**Salicylic acid-induced differential resistance to the *Tomato yellow leaf curl virus* among resistant and susceptible tomato cultivars**

**Tong Li, Ying Huang, Zhi-Sheng Xu, Feng Wang, Ai-Sheng Xiong**

State Key Laboratory of Crop Genetics and Germplasm Enhancement, Ministry of Agriculture and Rural Affairs Key Laboratory of Biology and Germplasm Enhancement of Horticultural Crops in East China, College of Horticulture, Nanjing Agricultural University, 1 Weigang, Nanjing, 210095, China

*Please address all correspondence to: A.S. Xiong ([xiongaisheng@njau.edu.cn](mailto:xiongaisheng@njau.edu.cn))

---------------

Dr. Ai-Sheng Xiong

Professor

State Key Laboratory of Crop Genetics and Germplasm Enhancement,

College of Horticulture,

Nanjing Agricultural University,

Nanjing, 210095, China

Fax: 86 25 84396790

Email: [xiongaisheng@njau.edu.cn](mailto:Xiongaisheng@njau.edu.cn)

**Additional file 1:**

**
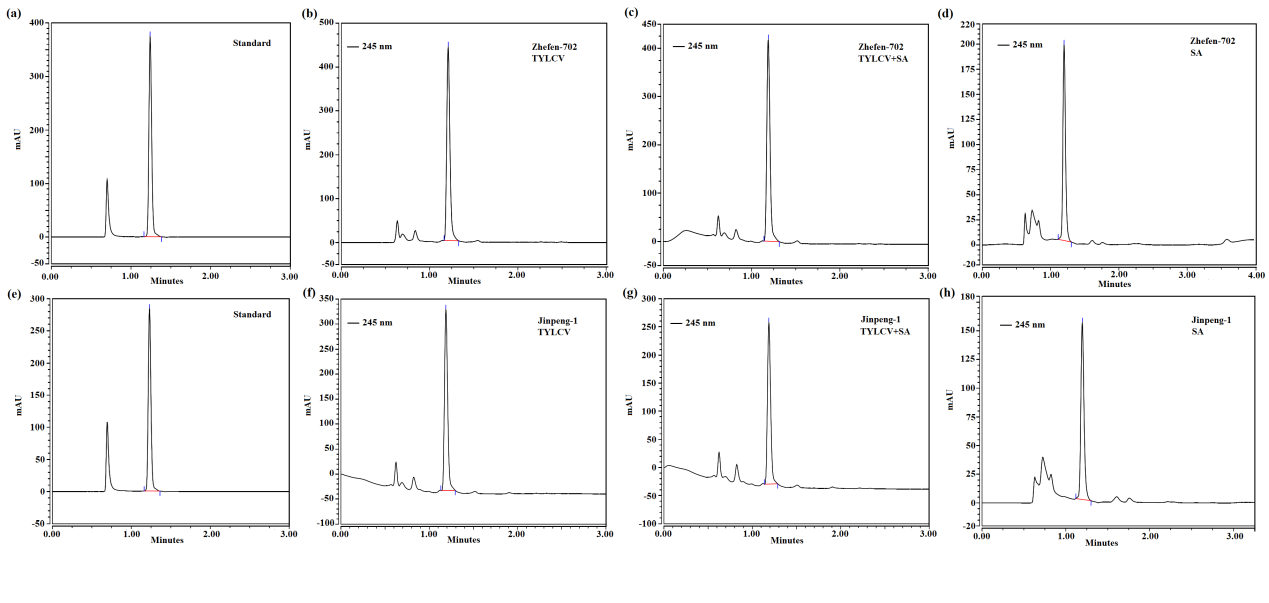
**

Figure S1 HPLC chromatogram of ascorbic acid (AsA) in two tomato cultivars at 4 days after infected with TYLCV

(a), (e) The standard curve of AsA. (b), (c), (d) The HPLC chromatogram of AsA in salicylic acid (SA), TYLCV, and SA+TYLCV treated plants in ‘Zhefen-702’ at 4 days post inoculated (dpi) with TYLCV.

(f), (g), (h) The HPLC chromatogram of AsA in SA, TYLCV, and SA+TYLCV treated plants in ‘Jinpeng-1’ at 4 dpi.


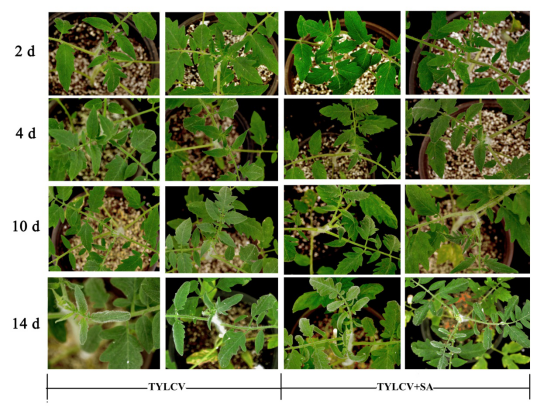


**Figure S2 Phenotype enlargement of SA+TYLCV and only TYLCV treated**

**plants in ‘Zhefen-702’ during whole experiment period.**


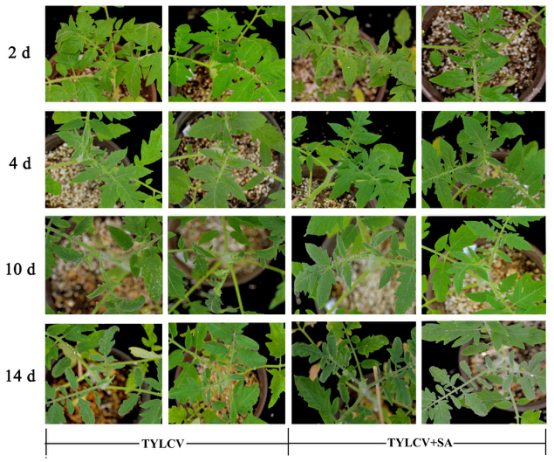


**Figure S3 Phenotype enlargement of SA+TYLCV and only TYLCV treated plants in ‘Jinpeng-1’ during whole experiment period**
